# The role of ethnicity, biological sex, and psychotropic agents in early and late onset Alzheimer’s disease

**DOI:** 10.3389/fnagi.2022.1052330

**Published:** 2022-12-22

**Authors:** Alyssa Miller, Ashna Desai, Laurie Theriot Roley, Richard L. Goodwin, Adebobola I. Nathaniel, Thomas I. Nathaniel

**Affiliations:** ^1^Department of Biology, North Greenville University, Tigerville, SC, United States; ^2^Department of Biology, University of South Carolina, Columbia, SC, United States; ^3^Department of Geriatric Medicine, Prisma Health, Greer, SC, United States; ^4^School of Medicine Greenville, University of South Carolina, Greenville, SC, United States

**Keywords:** Alzheimer’s disease, late onset of Alzheimer’s disease, early onset of Alzheimer’s disease, male, female

## Abstract

**Objective:**

This study investigates differences in pharmacological and demographic factors among male and female patients with Late-onset Alzheimer’s disease (LOAD) and Early-onset Alzheimer’s disease (EOAD).

**Method:**

Data are from 10,126 AD patients, 9,290 were diagnosed with LOAD, while 836 were diagnosed with EOAD. Data were collected from the Prisma Health Upstate Alzheimer’s patients’ registry between 2016 and 2021. The logistic regression analysis was used to assess the association between pharmacological and demographic factors in males and females with LOAD and EOAD.

**Results:**

In the adjusted analysis for males, patients that were administered memantine [odd ratio (OR) = 1.588, 95% CI, 1.175–2.145, *p* = 0.003], and buspirone [OR = 1.971, 95% CI, 1.221–3.183, *p* = 0.006] were more likely to be associated with EOAD, while increasing age [OR = 0.816, 95% CI, 0.799–0.834, *p* < 0.001] was associated with LOAD. Female patients with a history of alcohol (ETOH) use were more likely to be associated with EOAD while increasing age [OR = 0.845, 95% CI, 0.834–0.857, *p* < 0.001], treatment with memantine [OR = 0.774, 95% CI, 0.627–0.956, *p* = 0.017], African Americans [OR = 0.621, 95% CI, 0.462–0.835, *p* = 0.002] and tobacco use [OR = 0.529, 95% CI, 0.424–0.660, *p* < 0.001] were associated with LOAD.

**Conclusion:**

Our findings identified specific demographic and pharmacological factors associated with males and females with LOAD and EOAD. These findings suggest the need to develop strategies to eliminate disparity in the care of LOAD or EOAD patients.

## 1. Introduction

In the aging population, Alzheimer’s Disease (AD) is a common diagnosis, classified as a progressive neurodegenerative disorder (Kamboh, 2004) characterized by behavioral changes, cognitive deficits, and memory loss (Nebel et al., 2018). More than 5 million Americans are diagnosed with AD, while an estimated 5.3% of these patients are over 65 years old (Nebel et al., 2018), indicating that age is the most prominent risk factor for AD (Balin and Hudson, 2014; Cacace et al., 2016). Early Onset of Alzheimer’s Disease (EOAD) accounts for a diagnosis under the age of 65, while Late-Onset of Alzheimer’s disease (LOAD) is reported among those 65 years and older (Beam et al., 2018). Late-onset Alzheimer’s disease constitutes more than 85% of the total AD patients (Cacace et al., 2016). Risk factors associated with LOAD are vascular risk factors, sleep disorders, and traumatic brain injury (Rabinovici, 2019), while increased cognitive and physical activity throughout the lifespan reduces the risk of AD or LOAD. Typical LOAD symptoms begin with repeating statements or questions, misplacing items, and forgetting conversations (Rabinovici, 2019). As LOAD progresses, the patients may struggle with decision-making, getting lost in familiar areas, calculations, and forming sentences (Laws et al., 2016). The clinical features of LOAD include the inability to perform daily activities, visuospatial impairments, and executive dysfunction (Rabinovici, 2019). EOAD comprises 5–10% of AD diagnoses (Beam et al., 2018) and is often associated with a more aggressive disease progression (Kim et al., 2021). Most EOAD cases are diagnosed between the ages of 45 to 65 (Balin and Hudson, 2014; Subramaniapillai et al., 2021). Visual dysfunction, dyscalculia, apraxia, executive dysfunction, and aphasia are all common symptoms in addition to the expected memory impairments (Cacace et al., 2016).

Several factors, including demographic and pharmacological history, may be associated with the diagnosis of EOAD and LOAD, as well as treatment outcomes. For example, females are at a higher risk of developing AD than males (Mielke, 2018) because males are reported to attain higher cognitive development than females (Rabinovici, 2019). Moreover, the effect that a lifetime of accumulation of cognitive and social experiences has on brain function and cognitive performance, which is protective against AD, is higher in males than females (Subramaniapillai et al., 2021). In addition, females have less reserve, which leads to higher susceptibility to LOAD and EOAD and a faster decline in cognitive functions (Kim et al., 2021). In the AD population, females account for two-thirds of the cases (Beam et al., 2018) and are more likely to be predisposed to the risk factors leading to AD diagnosis than males (Beam et al., 2018). Hormonal changes and the rate at which the changes occur over the lifetime might play a significant role in the susceptibility to aging diseases, including LOAD or EOAD (Pike, 2017). For example, males diagnosed with AD show decreased levels of both circulating and brain levels of testosterone compared to males without AD (Li and Singh, 2014). While hormones, such as testosterone decline slowly as males age, females typically experience a sharp decline in sex hormones like estrogen at menopause which might be one of the contributing factors to a higher susceptibility of females to aging diseases, including LOAD and EOAD.

While there is no FDA-approved medication for AD, treatment options are focused on reducing neurologic deterioration to manage and slow down the progression of the disease (Li and Singh, 2014). Both EOAD and LOAD have the same treatment options. The use of cholinesterase inhibitors (ChEI), which are antagonists of a receptor for the neurotransmitter glutamate and drugs usually prescribed to combat depression and other illnesses (Isik, 2010), is the main option for therapy in AD cases that are considered mild to moderate for EOAD and LOAD (Wattmo and Wallin, 2017). Overall, cognitive response in patients treated with ChEIs showed better outcomes in patients diagnosed with LOAD than EOAD (Li and Singh, 2014). In contrast, administration of donepezil to patients diagnosed with EOAD showed a much slower deterioration when compared with patients diagnosed with LOAD (Wattmo and Wallin, 2017). Males are more likely to exhibit a short-term positive response than females when administered tacrine and galantamine (Bailey-Taylor et al., 2022), while older females diagnosed with LOAD were less likely to be administered ChEIs. In addition to pharmacological factors, demographic factors also play a role in treating EOAD and LOAD. For example, African American females with LOAD and a history of alcohol (ETOH) use treated with selective serotonin receptor inhibitors (SSRIs) were more likely to be given ChEIs (Bailey-Taylor et al., 2022). Moreover, EOAD patients are known to be younger and have a higher education level than those diagnosed with LOAD (Palasí et al., 2015).

More than two-thirds of people diagnosed with AD are reported to be females (Mielke, 2018). Although our sample is restricted to EOAD and LOAD, we assume that more females may be affected than males since this is typical in the AD population (Subramaniapillai et al., 2021). Therefore, we hypothesized that males and females with EOAD and LOAD differ in medications for treatments, including ChEIs, SSRIs, and SGAs. Since males and females differ in cognitive progression, with females declining at a much faster rate than males (Lin et al., 2015; Laws et al., 2016; Rabinovici, 2019), we determined specific demographic factors contributing to differences in biological sex among EOAD and LOAD patients who received ChEI, SSRIs, and SGAs. In other words, we determined whether the demographic and pharmacologic factors associated with EOAD, and LOAD differ among male and female patients.

## 2. Materials and methods

Data for EOAD and LOAD patients were extracted from the Alzheimer’s registry of Prisma Health-Upstate between February 2016 to August 2021. The approval for this study was obtained from the Prisma health committee for research compliance. Inclusion factors for this study were medication history, risk factors, and demographics. We extracted data for the patient’s medication history, including selective serotonin receptor inhibitors (SSRI), specifically citalopram, escitalopram, paroxetine, and central acetylcholinesterase inhibitors (ChEI), including donepezil, galantamine, and rivastigmine. We also extracted data for second-generation antipsychotics (SGA), including aripiprazole, olanzapine, risperidone, memantine, trazodone, buspirone, and valproate. Data for tobacco and alcohol use, race, biological, sex, age, and ethnicity were also collected.

### 2.1. Statistical analysis

All statistical data analyzes in this study were performed using IBM SPSS v.26 (IBM Corp., Armonk, NY, United States), and for all the analyzes, *p* < 0.05 was considered statistically significant. All continuous variables were analyzed and presented as means, and standard deviations, while categorical variables were presented as percentages. The student’s *t*-test was used to analyze continuous variables, while the chi-square test was used for categorical variables. Differences in demographic and pharmacologic factors were compared between the EOAD or LOAD patients using univariate and multivariate logistic regression models. They were presented as unadjusted and adjusted by sex, age, and risk factors. Our study is a non-randomized design. Therefore, a *post hoc* analysis was used to adjust for the demographic and pharmacologic factors associated with male or female patients with EOAD or LOAD.

The EOAD or LOAD categories were used for the regression model as the dependent variable. The independent variables were the pharmacological and demographic factors stratified by biological sex for EOAD or LOAD patients. Odds ratios (OR) with 95% confidence intervals (95% CIs) of outcome measures were obtained from these models. The odds ratio for patients with EOAD or LOAD was analyzed separately for males, females, and the entire population, independent of biological sex. In addition, multicollinearity and interactions were checked among independent variables using the Hosmer-Lemeshow test. Finally, the area under the receiver operating curve (AUROC), was determined to test the model’s sensitivity, specificity, and accuracy.

## 3. Results

Table 1 compares the demographic and pharmacological characteristics of early onset versus late onset AD. A total of 10,126 AD patients were identified in this study, out of which 9,290 were diagnosed with LOAD, while 836 were diagnosed with EOAD. As shown in Table 1, patients with EOAD were more likely to be younger (73.58 ± 11.42 vs. 86.28 ± 7.41), males (36.1% vs. 31.7%), African American (14.0% vs. 11.2%), and present with higher rates of ETOH use (24.3% vs. 13.2%). In addition, they were more likely to be taking a ChEI (68.9% vs. 63.3%), specifically galantamine (2.4% vs. 1.5%) and donepezil (62.1% vs. 55.0%). EOAD patients displayed higher rates of SGA use (22.8% vs. 16.0%), including olanzapine (7.1% vs. 4.3%), citalopram (14.5% vs. 11.9%), risperidone (15.8% vs. 10.8%), and aripiprazole (4.7% vs. 2.2%). More buspirone (11.6% vs. 7.2%) and memantine (53.2% vs. 44.9%) were used for EOAD compared with LOAD patients.

**Table 1:** Demographic and pharmacological characteristics of early-onset and late-onset Alzheimer’s disease patients.

Characteristic	Late onset	Early onset	
Number of patients	9,290	836	*p*-value
Age group: no. (%)				
<50	0 (0.0)	6 (0.7)	<0.001*^a^
50-59	0 (0.0)	68 (8.1)	
60–69	136 (1.5)	243 (29.1)	
70–79	1,638 (17.6)	272 (32.5)	
> = 80	7,516 (80.9)	247 (29.5)	
Mean ± SD	86.28 ± 7.41	73.58 ± 11.42	<0.001*^b^
Gender no (%)				
Male	2,949 (31.7)	302 (36.1)	0.009*^a^
Female	6,341 (68.3)	534 (63.9)	
Race: no (%)				
White	7,808 (84.0)	692 (82.8)	0.008*^a^
Black	1,036 (11.2)	117 (14.0)	
Other	446 (4.8)	27 (3.2)	
Hispanic ethnicity: no. (%)	179 (1.9)	16 (1.9)	0.98
ETOH	1,206 (13.2)	201 (24.3)	<0.001*^a^
Tobacco use	3,898 (42.9)	368 (44.3)	0.435
Length of stay	1.94 ± 4.80	1.91 ± 6.50	0.869
Medications				
Central acetylcholinesterase inhibitor	5,885 (63.3)	576 (68.9)	0.001*^a^
Donepezil	5,113 (55.0)	519 (62.1)	<0.001*^a^
Galantamine	139 (1.5)	20 (2.4)	0.046*^a^
Rivastigmine	1,124 (12.1)	118 (14.1)	0.089
Second generation antipsychotic	1,487 (16.0)	191 (22.8)	<0.001*^a^
Aripiprazole	209 (2.2)	39 (4.7)	<0.001*^a^
Olanzapine	398 (4.3)	59 (7.1)	<0.001*^a^
Risperidone	999 (10.8)	132 (15.8)	<0.001*^a^
Selective serotonin receptor inhibitor	3,066 (33.0)	295 (35.3)	0.179
Citalopram	1,105 (11.9)	121 (14.5)	0.029*^a^
Escitalopram	2018 (21.7)	193 (23.1)	0.361
Paroxetine	0 (0.0)	0 (0.0)	
Memantine	4,168 (44.9)	445 (53.2)	<0.001*^a^
Trazadone	0 (0.0)	0 (0.0)	
Buspirone	671 (7.2)	97 (11.6)	<0.001*^a^
Valproate	0 (0.0)	0 (0.0)	

Results for the continuous variables are presented as Mean ± SD, while discrete data are presented as percentage frequency. Pearson’s Chi-Square compares differences between demographic and clinical characteristics in patients with Alzheimer’s disease stratified by early or late onset.

^a^Pearson’s Chi-Squared test; ^b^Student’s *t* test; *p-value < 0.05.

As shown in Table 2 2,949 males presented with LOAD, while 302 were diagnosed with EOAD. Patients with EOAD were more likely to be Hispanics (3.4% vs. 1.1%) and younger (71.76 ± 11.37 vs. 85.59 ± 7.15). In addition, this group showed higher rates of ETOH use (28.6% vs. 18.2%), higher usage of ChEIs (77.5% vs. 64.5%), including galantamine (4.0% vs. 2.1%), and donepezil (66.6% vs. 56.2%). EOAD group also showed higher use of SSRIs (33.8% vs. 27.3%), including aripiprazole (2.6% vs. 1.2%), buspirone (9.3% vs. 5.8%) and memantine (63.2% vs. 47.0%). Six thousand, three hundred forty-one females were diagnosed with LOAD, while 534 were diagnosed with EOAD. Females with EOAD were more likely to be younger (74.60 ± 11.33 vs. 86.60 ± 7.51), African Americans (15.0% vs. 15.2%), and presented with higher rates of ETOH usage (21.9% vs. 10.9%). Females EOAD patients were more likely to be treated with donepezil (59.6% vs. 54.5%), SGAs (26.4% vs. 16.0%), including aripiprazole (5.8% vs. 2.8%), risperidone (17.4% vs. 10.7%), and olanzapine (8.1% vs. 4.2%). They were also likely to be treated with SSRIs, including citalopram (17.4% vs. 12.9%) and buspirone (12.9% vs. 7.9%).

**Table 2:** Demographic and pharmacological characteristics of early versus late-onset Alzheimer’s disease patients stratified by biological sex.

	Male			Female		
Characteristic	Late onset	Early onset		Late onset	Early onset	
Number of patients	2,949	302	*p*-value	6,341	534	*p*-value
Age group: no. (%)							
<50	0 (0.0)	6 (2.0)	<0.001*^a^	0 (0.0)	0 (0.0)	<0.001*^a^
50-59	0 (0.0)	40 (13.2)		0 (0.0)	28 (5.2)	
60–69	32 (1.1)	71 (23.5)		104 (1.6)	172 (32.2)	
70–79	562 (19.1)	117 (38.7)		1,076 (17.0)	155 (29.0)	
> = 80	2,355 (79.9)	68 (22.5)		5,161 (81.4)	179 (33.5)	
Mean ± SD	85.59 ± 7.15	71.76 ± 11.37	<0.001*^b^	86.60 ± 7.51	74.60 ± 11.33	<0.001*^b^
Race: no (%)							
White	2,590 (87.8)	251 (83.1)	0.051	5,218 (82.3)	441 (82.6)	0.006*^a^
Black	246 (8.3)	37 (12.3)		790 (12.5)	80 (15.0)	
Other	113 (3.8)	14 (4.6)		333 (5.3)	13 (2.4)	
Hispanic ethnicity: no. (%)	31 (1.1)	10 (3.4)	0.001*^a^	148 (2.3)	6 (1.1)	0.067
ETOH	526 (18.2)	86 (28.6)	<0.001*^a^	680 (10.9)	115 (21.9)	<0.001*^a^
Tobacco use	1879 (65.5)	184 (61.5)	0.172	2019 (32.5)	184 (34.7)	0.313
Length of stay	2.13 ± 6.88	1.87 ± 3.68	0.514	1.84 ± 3.42	1.93 ± 7.65	0.805
Medications							
Central acetylcholinesterase inhibitor	1902 (64.5)	234 (77.5)	<0.001*^a^	3,983 (62.8)	342 (64.0)	0.572
Donepezil	1,657 (56.2)	201 (66.6)	0.001*^a^	3,456 (54.5)	318 (59.6)	0.024*^a^
Galantamine	62 (2.1)	12 (4.0)	0.038*^a^	77 (1.2)	8 (1.5)	0.569
Rivastigmine	342 (11.6)	37 (12.3)	0.736	782 (12.3)	81 (15.2)	0.057
Second generation antipsychotic	470 (15.9)	50 (16.6)	0.78	1,017 (16.0)	141 (26.4)	<0.001*^a^
Aripiprazole	34 (1.2)	8 (2.6)	0.028*^a^	175 (2.8)	31 (5.8)	<0.001*^a^
Olanzapine	131 (4.4)	16 (5.3)	0.495	267 (4.2)	43 (8.1)	<0.001*^a^
Risperidone	318 (10.8)	39 (12.9)	0.259	681 (10.7)	93 (17.4)	<0.001*^a^
Selective serotonin receptor inhibitor	806 (27.3)	102 (33.8)	0.017*^a^	2,260 (35.6)	193 (36.1)	0.816
Citalopram	286 (9.7)	28 (9.3)	0.811	819 (12.9)	93 (17.4)	0.003*^a^
Escitalopram	529 (17.9)	63 (20.9)	0.21	1,489 (23.5)	130 (24.3)	0.652
Paroxetine	0 (0.0)	0 (0.0)		0 (0.0)	0 (0.0)	
Memantine	1,385 (47.0)	191 (63.2)	<0.001*^a^	2,783 (43.9)	254 (47.6)	0.1
Trazadone	0 (0.0)	0 (0.0)		0 (0.0)	0 (0.0)	
Buspirone	170 (5.8)	28 (9.3)	0.015*^a^	501 (7.9)	69 (12.9)	<0.001*^a^
Valproate	0 (0.0)	0 (0.0)		0 (0.0)	0 (0.0)	

Results for the continuous variables are presented as Mean ± SD, while discrete data are presented as percentage frequency. Pearson’s Chi-Square is used to compare differences between demographic and clinical characteristics in groups with early versus late-onset Alzheimer’s disease based on biological sex.

^a^Pearson’s Chi-Squared test; ^b^Student’s *t* test; *p-value < 0.05.

**Figure 1:**
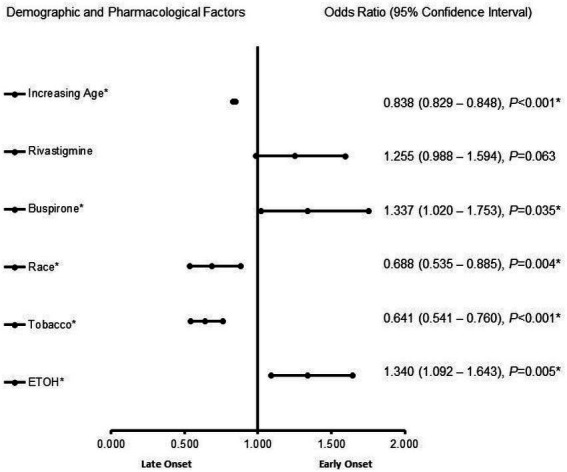
Demographic and pharmacological factors associated with early onset Alzheimer’s disease compared to late-onset Alzheimer’s disease independent of biological sex. Adjusted OR < 1 denotes factors that are associated with a late onset, while OR > 1 denotes factors that are associated without an early onset. Hosmer-Lemeshow test (*p* < 0.001*), Cox & Snell (*R^2^* = 0.148). The overall classified percentage of 93.4% was applied to check for the fitness of the regression model. *Indicates statistical significance (*p* < 0.05) with a 95% confidence interval.

Figure 1 presents demographic and pharmacological factors associated with early-onset Alzheimer’s disease compared to late-onset Alzheimer’s disease independent of biological sex. In the adjusted analysis, ETOH (1.340, 95% CI, 1.092–1.643, *p* = 0.005) and buspirone (OR = 1.255, 95% CI, 0.988–1.594, *p* = 0.035) were associated with EOAD, while tobacco use (0.641, 95% CI, 0.541–0.760, *p* < 0.001), race (0.688, 95% CI, 0.535–0.885, *p* = 0.004), and increasing age (0.838, 95% CI, 0.829–0.848, *p* < 0.001) were associated with LOAD (Figure 1). The model was strong, as shown by the ROC curve, with the area under the curve (AUROC) = 0.820 (95% CI, 0.801–0.839, *p* < 0.001).

**Figure 2:**
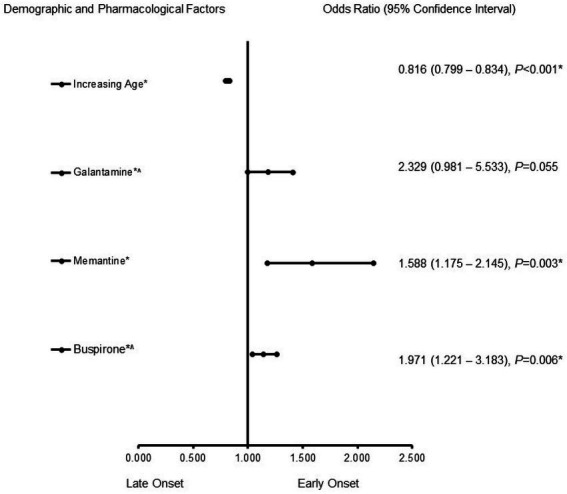
Demographic and pharmacological risk factors associated with early-onset Alzheimer’s disease in males. Adjusted OR < 1 denotes factors associated with a -onset, while OR > 1 denotes factors associated with an early onset. Hosmer-Lemeshow test (*p* < 0.001*), Cox & Snell (*R*^2^ = 0.187). The overall classified percentage of 93.3% was applied to check for the fitness of the regression model. The asterisk (*) Indicates statistical significance (*p* < 0.05) with a 95% confidence interval.

In the male patients with AD (Figure 2), memantine (OR = 1.588, 95% CI, 1.175–2.145, *p* = 0.003) and buspirone (OR = 1.971, 95% CI, 1.221–3.183, *p* = 0.006) were more likely to be associated with EOAD, whereas increasing age (OR = 0.816, 95% CI, 0.799–0.834, *p* < 0.001) was associated more with LOAD. The predictive capability of the logistic regression was strong, as shown by the area under the curve (AUROC), which is 0.856 (95% CI, 0.829–0.883, *p* < 0.001).

**Figure 3:**
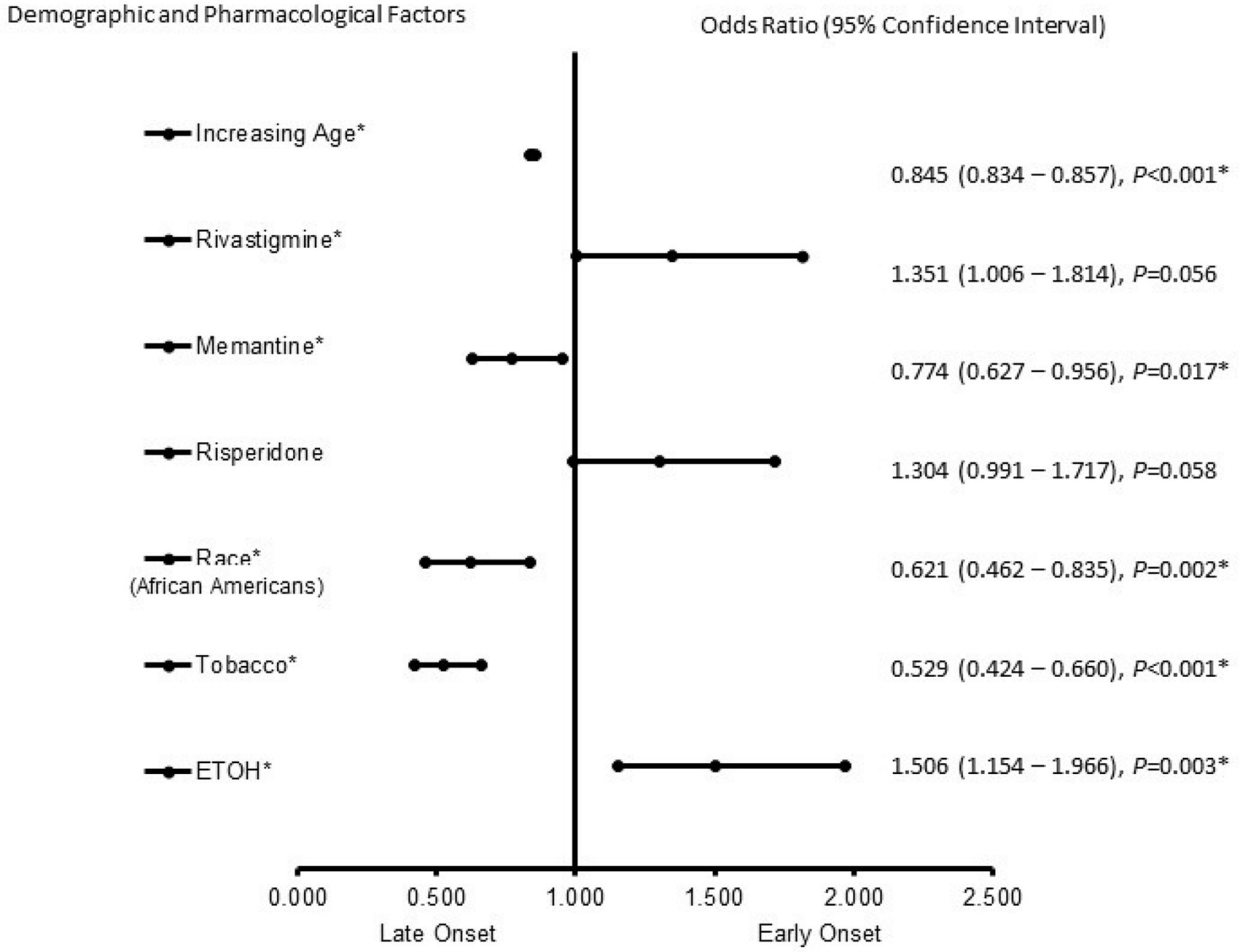
Demographic and Pharmacological risk factors associated with early-onset Alzheimer’s disease in female patients. Adjusted OR < 1 denotes factors associated with a late onset, while OR > 1 denotes factors associated with early onset. Hosmer-Lemeshow test (*p* < 0.001*), Cox & Snell *(R^2^* = 0.132). The overall classified percentage of 93.3% was applied to check for the fitness of the logistic regression model. The asterisk (*) Indicates statistical significance (*p* < 0.05) with a 95% confidence interval.

In females (Figure 3), ETOH use (OR = 1.506, 95% CI, 1.154–1.966, *p* = 0.003) was more likely to be associated with EOAD, whereas increasing age (OR = 0.845, 95% CI, 0.834–0.857, *p* < 0.001), memantine (OR = 0.774, 95% CI, 0.627–0.956, *p* = 0.017), race [(African Americans) (OR = 0.621, 95% CI, 0.462–0.835, *p* = 0.002)] and tobacco use (OR = 0.529, 95% CI, 0.424–0.660, *p* < 0.001) were associated with LOAD. The power of the logistic regression was strong, as shown by the area under the curve (AUROC), which is 0.808 (95% CI, 0.784–0.833, *p* < 0.001).

## 4. Discussion

Alzheimer’s disease has a misleading onset making it occasionally difficult to separate the disease condition from an age-related deterioration (DeTure and Dickson, 2019). Diagnosis of AD before the age of 65 years is described as presenting with EOAD (Borges et al., 2018), while those with an onset of AD after 65 years of age are described as having LOAD (Vardarajan et al., 2014). While EOAD is often associated with a more aggressive disease progression (Kim et al., 2021), whether there are differences in demographic and other pharmacological factors in males and females LOAD and EOAD patients is not fully understood. The current study determined differences in biological sex among LOAD and EOAD patients treated with ChEIs, SGAs, and SSRIs medications.

In the adjusted analysis, male patients treated with memantine and buspirone were more likely to be associated with EOAD, while those with increasing age were more likely to be associated with LOAD. In addition, female patients with increasing age were more likely to be associated with EOAD. In contrast, EtOH, tobacco, treatment with memantine, and African American females were associated more with LOAD.

Pharmacological management of AD currently includes using ChEIs, such as galantamine, donepezil, and rivastigmine, that improve cognitive functions (Wilkinson et al., 2004). Therefore, the decision to treat LOAD or EOAD patients with a ChEI is based on the possibility that AD was the underlying etiology (Wattmo and Wallin, 2017; Grossberg et al., 2019), indicating that other medications, such as SGAs and SSRIs, may be combined with a ChEI for the treatment of symptoms other than those found in LOAD or EOAD patients.

We observed that more females or males with EOAD and LOAD were more likely to be treated with memantine an SSRI medication. SSRIs selectively target the solute carrier family 6 members 4 responsible for terminating the action of serotonin in the synaptic cleft, consequently increasing this neurotransmitter availability at the synapse (Mdawar et al., 2020). Memantine is an SSRI that acts as both a dopamine agonist and a non-competitive NMDA receptor antagonist and is utilized in mild to moderate AD (Liu et al., 2019). In addition, memantine also acts on ion channel receptors of cholinergic and serotonergic systems, supporting learning processes and memory (Rammes et al., 2008). Therefore, memantine is often used in addition to ChEI therapy; the combination of the medications is more effective in cognitive functions than isolated use (Rammes et al., 2008). Overall, patients taking memantine exhibited less decline in memory and language, as well as a decrease in agitation and aggression (Fox et al., 2012). The faster rates of behavioral and cognitive decline and predominance of atypical forms in EOAD suggest that neuropsychiatric and behavioral symptoms could be different in EOAD compared to LOAD. The differential patterns of behavioral symptoms observed between EOAD, and LOAD indicate a pattern of selective vulnerability extending to the brain’s subcortical structures. Irritability, agitation, aggression, and sleep disturbances are common in AD (Lyketsos et al., 2011). Previous studies suggest that these symptoms increase in severity across the disease course for EOAD and LOAD, irrespective of the biological sex (Ehrenberg et al., 2018). Therefore, the use of memantine for both males and females with EOAD and LOAD in the current study is not surprising, as memantine has a beneficial effect on the behavioral symptoms of patients with moderate to severe AD, with the most pronounced effect on agitation and aggression.

Male EOAD patients were treated with buspirone, an anxiolytic medication commonly used to treat AD. Buspirone has anti-aggressive properties that aid in diagnosing AD, particularly EOAD, with an average of 40% of patients displaying a positive outcome when administered buspirone (Cooper, 2003). Our finding that male EOAD patients were treated with buspirone suggests future studies on the role of buspirone in managing agitation and behavioral disturbances associated with EOAD.

We found that increasing age was strongly associated with LOAD in males and females. In ages over 65, the prevalence of an AD diagnosis increases by 19% (Qiu et al., 2009). In both males and females, as aging occurs, there is a reduction of brain volume and weight, loss of synapses and dendrites, and an enlargement of ventricles (Zia et al., 2021). These all lead to decreased cognitive function associated with AD (Zia et al., 2021). In addition, as an individual age increases, myelin begins to break down, affecting the white matter tracts and leading to the development of LOAD (Stahon et al., 2016). Similarly, loss of cells in the brain stem, specifically the locus coeruleus (LC), is common in aging patients (Beardmore et al., 2021). This loss of cells impairs the blood–brain barrier (BBB), indicating age-related vascular factors in diagnosing LOAD in males and females AD patients (Banks et al., 2021).

The clinical manifestation of AD is reported to be different for African Americans compared to non-Hispanic whites. We observed that African American females were associated with LOAD. African Americans frequently present at an earlier age of onset and exhibit greater severity of symptoms at the time of presentation (Chen and Zissimopoulos, 2018). This is consistent with the fact that compared to non-Hispanic whites, African Americans are less likely to seek medical attention, and when they do, they present later in the disease course. A growing body of evidence suggests that African Americans are less likely than non-Hispanic whites to receive Alzheimer’s treatment, such as ChEI therapy or memantine (Gilligan et al., 2012). Our current finding that African American females were associated with LOAD contributes to the existing literature on AD among African Americans. In addition, this finding provides information for understanding LOAD among other racial and ethnic groups.

Tobacco use was associated with females with LOAD. The neurological effects of tobacco occur when an individual has been using the substance for an extended period, which is reported to have a stronger association with LOAD than EOAD (Mdawar et al., 2020). Moreover, individuals over 65 with a history of tobacco use demonstrated declines in memory, learning, executive functions, and processing speed compared to patients without tobacco use (Sabia et al., 2012). In females with a history of tobacco use, there is a higher average density of neuritic plaques found in the neocortex, entorhinal cortex, and hippocampus and a higher density of neurofibrillary tangles (Durazzo et al., 2014). This difference was not observed in males with a history of tobacco use (Durazzo et al., 2014). A future study investigates the association between LOAD and tobacco use in females will help establish the relationship between neurofibrillary tangles and LOAD in female patients with tobacco use.

Females with ETOH use were more likely to be associated with EOAD. Recent studies indicate that alcohol plays a much more significant role in EOAD than previously thought, as mild–moderate alcohol intake is widely associated with a lower risk of AD (Neafsey and Collins, 2011; Sabia et al., 2018). At the same time, heavy drinking increases the risk (Heymann et al., 2016). Some studies have suggested that the no alcohol and heavy drinking conveying an increased risk of developing AD compared to moderate drinking, indicates a ‘U shaped’ relationship (Heymann et al., 2016; Koch et al., 2019). There is debate about whether the effects of alcohol on AD are due to ethanol itself or if a specific beverage type biases these results. Several studies have found that wine, not beer or hard liquor, is protective against AD development (Neafsey and Collins, 2011; Heymann et al., 2016), but another study disagreed with this finding (Ruitenberg et al., 2002). One study found that mixed drinks are solely beneficial (Weyerer et al., 2011), while others found that beer (Truelsen et al., 2002) or spirits are associated with worse outcomes (Sabia et al., 2014). Although, little is known about the mechanisms and how each beverage type might affect AD progression. Our finding reveals that ETOH use was more likely to be associated with females with EOAD. Further investigation into how different alcohol types and drinking habits could alter the course of AD in female patients with EOAD may shed more light on the mechanisms and relationship between female patients and EOD.

### 4.1. Limitations

This is a retrospective study, and potential limitations must be considered before interpreting the results. The data utilized in this study was collected from a single Alzheimer’s registry, so it cannot be extrapolated to other institutions. Since data were collected from electronic medical records, human errors should also be considered factors and the potential for patients to be excluded. This study analyzed 10,126 patients; this comprises a very small percentage compared to the number of AD cases worldwide. Due to the limited number of patients in this study and the inclusion of more females than males, the results may not represent similar studies on a broader scale. In addition, alcohol use in the analysis did not consider the type of alcohol consumed, whether wine, beer, and liquor, the average amount consumed, or frequency of intake. The socio-economic status and other habits such as drug abuse was not included in our database for analysis. There is also the possibility of one race being represented more than other among subjects used in this study or specific age ranges showing higher levels of inclusion. In future studies, a larger population size from multiple institutions could provide more insight into pharmacological and demographic factors associated with males and females diagnosed with EOAD and LOAD.

## 5. Conclusion

There are similarities and differences in demographic and pharmacological factors associated with males and females with LOAD and EOAD. The significance of this study lies in the opportunity to determine different medications used in treating males and female LOAD and EOAD patients. In addition, this study’s findings support further investigation into developing strategies to eliminate disparity in the care of LOAD or EOAD patients.

## Data availability statement

The original contributions presented in the study are included in the article/supplementary material, further inquiries can be directed to the corresponding author.

## Ethics statement

The institutional review board approved this study of PRISMA Health institutional committee for ethics (approval number: 00052571). This is a retrospective data collection. All data were fully anonymized before they were accessed. Written informed consent for participation was not required for this study in accordance with the national legislation and the institutional requirements.

## Author contributions

AM, AD, LR, RG, AN, and TN designed the concept, experiment, and data analysis. RG, AN, and TN critically revised the drafts, interpreted the results, read and approved the last version of this manuscript. All authors have read and approved the manuscript.

## Funding

This study was funded by NIH R25 Grant. The grant # is 5R25AG067934.

## Conflict of interest

The authors declare that the research was conducted in the absence of any commercial or financial relationships that could be construed as a potential conflict of interest.

## Publisher’s note

All claims expressed in this article are solely those of the authors and do not necessarily represent those of their affiliated organizations, or those of the publisher, the editors and the reviewers. Any product that may be evaluated in this article, or claim that may be made by its manufacturer, is not guaranteed or endorsed by the publisher.

## Abbreviations

AD: Alzheimer’s disease
AD: EOAD; Early onset of Alzheimer’s disease
LOAD: Late onset of Alzheimer’s disease
BBB: Blood–brain barrier
BPSD: behavioral and psychological symptoms of dementia
ETOH: Alcohol
IRB: Institutional Review Board
ChEI: Cholinesterase inhibitor
LC: Locus coeruleus
SGAs: Second-generation antipsychotics
SSRIs: Selective serotonin receptor inhibitor
ICD: International classification of diseases
MMSE: mini mental exam
AUROC: Area under the Receiver Operating Characteristics
NMDA: N-Methyl-D-Aspartate
ROC: Receiver operating characteristic curve
OR: Odd ratio
VIF: Variance Inflation factor
SPSS: Statistical Package for the Social Sciences software
SD: Standard deviations: 5-HT receptors; 5-hydroxytryptamine receptor
